# Prolactin and oxytocin as modulators of intestinal contractility and glucose uptake

**DOI:** 10.3389/fphys.2026.1703887

**Published:** 2026-02-26

**Authors:** Perla Alejandra Figueroa-Carrasco, Aída Jimena Velarde-Salcedo, Carmen Gonzalez

**Affiliations:** Facultad de Ciencias Químicas, Universidad Autónoma de San Luis Potosí, San Luis Potosí, San Luis Potosí, Mexico

**Keywords:** nitric oxide, oxytocin, prolactin, small intestine, smooth muscle

## Abstract

**Introduction:**

Prolactin (PRL) and oxytocin (OT) are bioactive hormones naturally present in maternal milk that support neonatal development, contribute to immune regulation and gut maturation in infants, and promote growth and cell differentiation in the small intestine. However, the individual and combined roles of these hormones in intestinal function remain unclear. This study aims to elucidate the physiological effects of PRL and OT on intestinal motility, nitric oxide (NO) production, and glucose uptake to better understand their influence during early development.

**Methods:**

Precontracted intestinal segments were placed in physiological solution, connected to isometric transducers, and exposed to various concentrations of PRL, OT, or PRL + OT, and changes in contractile responses were recorded. Glucose uptake was measured using everted sacs, and NO production was measured via the Griess method. PRL, OT, and PRL + OT modulated intestinal contractile activity, with effects varying by segment. OT induced higher NO levels than PRL at cumulative concentrations.

**Results and discussion:**

A single concentration of PRL or OT mostly preserved the contraction vs. % of the maximal contraction induced by KCl (100%), while PRL + OT reduced it and NO production in the duodenum and jejunum, but not in the ileum. Individually, PRL and OT increased glucose uptake, while their combination inhibited it, suggesting a modulatory mechanism regulating nutrient absorption. These findings support the role of PRL and OT as maternal milk-derived regulators of intestinal functions.

## 1 Introduction

The World Health Organization (WHO) has stated that breast milk is the best food for infants, as it is both safe and clean. It meets the infant’s nutritional needs during the first and second years of life, helping protect against common illnesses. Breast milk is a natural and complete food that contains antibodies that boost the immune system (WHO, 2024). It provides essential nutrients, including vitamins, minerals, fats, carbohydrates, proteins, enzymes, and hormones, that regulate digestion processes and growth factors that support healthy development. Additionally, breastfeeding provides calm, security, and comfort to both the infant and the mother (Modak et al., 2023).

Prolactin (PRL) is a 23-kDa peptide hormone released by the pituitary gland in response to nipple stimulation during breastfeeding. It supports the growth and multiplication of milk-producing cells in the mammary glands, thereby increasing breast milk production. PRL mixes with breast milk and reaches the baby’s digestive tract (Buj et al., 2002; Gopalakrishnan et al., 1981; Purkiewicz et al., 2025). It is essential for regulating calcium metabolism during pregnancy and lactation, as it stimulates renal calcium reabsorption and transcellular calcium absorption in the small intestine, thereby managing vital processes such as peristalsis (Nakkrasae et al., 2010). PRL likely plays a role in nutrient absorption and influences the paracellular movement of ions and small molecules in other tissues, such as lactose through the mammary epithelium, which is associated with mechanisms responsible for modifying the composition of the milk’s aqueous phase (Linzell et al., 1975). Additionally, PRL can be incorporated into neonatal nutrition as a supplement to promote the growth and development of the small intestine (Naim, 2010). This application highlights its potential as a functional ingredient in early-life dietary interventions to enhance intestinal maturation and long-term gastrointestinal health.

The small intestine is an organ that absorbs calcium into the body and expresses the PRL receptor (PRLR) mRNA, especially in the duodenum and jejunum segments. PRL considerably increases paracellular calcium transport and is responsible for adaptive changes in the intestinal mucosa, increasing the surface area for absorption and boosting the expression of calcium transporters in lactating rats (Charoenphandhu and Krishnamra, 2007; Dorkkam et al., 2013; Wongdee et al., 2016).

Oxytocin (OT) is a nonapeptide produced in hypothalamic–hypophyseal neurons, which stimulates oxytocin receptors (OTRs) to trigger milk ejection and parturition in response to sensory and emotional stimulation. This hormone causes the contraction of the mammary gland ducts, facilitating milk flow during breastfeeding and fostering a strong emotional bond between mother and child (Okumura et al., 2022). Oxytocin is also involved in the homeostasis of food intake and energy metabolism. These metabolic effects promote its therapeutic potential for treating chronic diseases such as obesity (Hong et al., 2021). OT regulates gastrointestinal (GI) motility, secretion, blood flow, cell turnover, and the release of neurotransmitters like nitric oxide (NO) (Melis et al., 1997) and peptides such as secretin (Takayanagi et al., 2017). OT also exhibits anti-secretory and anti-ulcer properties, aiding in wound healing and modulating immune and inflammatory processes (Asad et al., 2001; Li et al., 2015). OTRs are located in the gut and dorsal root ganglion neurons (Li et al., 2015; Okumura et al., 2022). They are concentrated in specific regions of the GI tract, such as the apical adherent junctions of enterocytes at the crypt–villus junction of the rat duodenum (Welch et al., 2009), as well as in the mucosa and smooth muscle of the stomach, duodenum, jejunum, and ileum in lactating and post-weaning rats (Huang et al., 2008).

When the baby ingests breast milk containing PRL and OT, these hormones may influence the maturation of the gastrointestinal tract during and after pregnancy. Studies suggest that both hormones may play a role in regulating several GI functions, such as motility and absorption (Abramovich et al., 1979; Lobie et al., 1993; Okumura et al., 2022).

NO is a free radical and a key mediator involved in GI function, known for its role in relaxing smooth muscle and regulating intestinal motility. It is produced from the amino acid L-arginine by the nitric oxide synthase (NOS) enzyme group (Jobgen et al., 2006). Because of the complex interplay between these hormones and NO, it is important to examine how PRL and OT can regulate contractile processes and glucose uptake in the small intestine by modulating NO production. There are three types of NOS: neuronal NOS (nNOS) and endothelial NOS (eNOS), which help sustain the body’s homeostasis, and inducible NOS (iNOS), which is highly expressed in inflammatory cells in response to immunogenic stimuli (Jobgen et al., 2006; Kamalian et al., 2020).

NO has dual effects on smooth muscle cells, particularly those in blood vessels and the GI system. At physiological concentrations [picomolar (pM)], NO promotes muscle relaxation, regulating intestinal peristalsis, gastric emptying, and antral motor activity. However, at increased concentrations, NO has been linked to various GI tract conditions, including peptic ulcers, gastritis, and cancer (Martin et al., 2001). *In vitro* studies demonstrated that the addition of sodium nitrite to the serosal region of the mouse ileum produced an absorptive effect. For example, NO’s possible role in regulating intestinal ion transport was studied in isolated sheets of mouse ileum mounted in Ussing flux chambers (Rao et al., 1994). Moreover, NOS inhibition increased serotonin-induced chloride secretion in the guinea pig distal colon *in vitro* and markedly decreased serotonin-induced diarrhea in mice (Lanas, 2008; Mourad et al., 1999). Potential sources of NO in the intestine include the endothelial cells of the capillaries that supply the small intestine, intrinsic intestinal tissue (mast cells, epithelium, smooth muscle, and neurons), resident and infiltrating leukocytes (neutrophils and monocytes), luminal gastric nitrate reduction, and, to a lesser extent, denitrification by commensal intestinal bacteria. NOS has been localized in myenteric and submucosal neurons of the subepithelial compartment and lamina propria, including submucosal arterioles and venules, as well as in apical epithelial cells (Mourad et al., 1999). These studies have demonstrated the effects of NO on the gut in maintaining homeostasis and preserving health.

To date, there have been insufficient studies focusing on the effects of PRL and OT, as well as their combination, on small intestine contractile regulation, their association with nutrient absorption (such as glucose), and whether they are related to NO production. This work aims to evaluate the impact of PRL, OT, and their combination using intestinal segments (duodenum, jejunum, and ileum) in an isolated young rat small intestine in an *ex vivo* model, evaluating the physiological profile upon the intestinal contraction, the association with the uptake of glucose, and whether these physiological actions are mediated by NO. Understanding how these hormones affect intestinal function, maturation, and absorption may also have substantial implications for the digestive health of infants, benefiting their development and overall wellbeing, and may provide insight into early-life intestinal physiology, with possible translational relevance.

## 2 Materials and methods

### 2.1 Chemicals

Recombinant human prolactin (PRL) was provided by Thermo Fisher Scientific Inc. (Waltham, MA, United States); OT and salts for physiological solutions were obtained from Merck KGaA (Darmstadt, Germany).

### 2.2 Experimental biomodels

Male young Wistar rats (biomodels), weighing 100–140 g, were housed in each treatment group. They were kept under a 12-h light and 12-h dark cycle with free access to water and food. For small bowel resection, the biomodels were anesthetized with sodium pentobarbital (50 mg/kg, injected intraperitoneally). Depth anesthesia was confirmed by the absence of the foot, ear, and tail pinch reflexes and decreased heart rate, and then the biomodels were euthanized by heart removal. The dissection of the small intestine segments (duodenum, jejunum, and ileum) was performed immediately. All procedures were conducted in accordance with institutional and international ethical guidelines for the Care and Use of Laboratory Animals of the National Institute of Health (NIH, publication No. 92-3415 Bethesda, MD, United States, 1992), and under the approved protocols (CEID 2020-012, CEID 2023-05-R1), submitted to the CONBIOÉTICA-24-CEI-003-20190726 at Facultad de Ciencias Químicas-Universidad Autónoma de San Luis Potosí, México.

### 2.3 Isolated rat small intestine ring contraction force measurement

To determine the tension of isolated rat small intestine rings, 1-cm segments from different portions of the intestine (duodenum, jejunum, and ileum) were cross-sectioned after the removal of the peripheral adipose tissue and placed in a Petri dish with Tyrode’s solution at pH 7.4 °C and 37 °C. The individual rings were mounted in physiological baths containing aerated Tyrode’s solution [136.9 mM NaCl, 2.69 mM KCl, 1.05 mM MgCl_2_, 1.8 mM CaCl_2_, 5.55 mM dextrose (glucose), 0.42 mM NaH_2_PO_4_, and 11.9 mM NaHCO_3_] at pH 7.4 and 37 °C, coupled to isometric transducers, and a passive load of 0.5 g, or 4.9 mN was applied. The bowel segments were stabilized for half an hour. Subsequently, they were precontracted with 30 mM KCl, which represents 100% of contraction, and the administration of either single or increasing cumulative concentrations of the hormones under study was conducted, ranging from 0.01 nM to 1.0 nM (Arrowsmith et al., 2018). Stabilized responses were measured at 29 min, 49 min, 69 min, and 89 min for cumulative additions. For single applications, stabilized responses were first evaluated at 29 min following KCl-induced precontraction. The hormonal treatment was then added, and its stabilized effect was quantified 20 min later (49 min). For each treatment, the stable plateau was defined as the final 3 min of the recording. Within this plateau, nine consecutive tension values were sampled at 20-s intervals and averaged to obtain a single stabilized value. Transient peaks occurring immediately after hormone addition were deliberately excluded from the analysis. The obtained data were collected and analyzed using LabScribe software (Covina, California, United States), and changes in tension were recorded in mN (Chávez-Hernández et al., 2024).

### 2.4 Determination of NO production

After evaluating intestinal contractility under various treatments, NO production was assessed by collecting aliquots of Tyrode’s solution bathing isolated intestinal rings (1 cm segments) for the duodenum, jejunum, and ileum. NO production was quantified indirectly through the measurement of nitrite (NO_2_^−^) and nitrate (NO_3_^−^) levels using the Griess reaction, as previously described (Miranda et al., 2001), based on the method outlined by Bryan and Grisham (2007). The organ bath contained 15 mL of Tyrode’s solution, and tissues were incubated for 20 min following PRL and/or OT addition before sample collection. NO_2_^−^/NO_3_^−^ concentrations were expressed as µM. Absorbance was measured at 595 nm, and concentrations were calculated from a standard curve ranging from 1 µM to 200 µM using an iMark microplate reader (Bio-Rad, Hercules, CA, United States) (Bryan and Grisham, 2007; Miranda et al., 2001).

### 2.5 Glucose uptake determination in everted intestinal sacs

Glucose uptake was assessed in everted jejunal sacs obtained from juvenile rats. From each animal, four sacs were prepared and independently assigned to the control, PRL, OT, or PRL + OT condition (one sac per condition per animal). Here, glucose uptake is defined as the relative decrease in mucosal glucose concentration, used as a functional estimate of intestinal glucose handling.

For the everted intestinal preparation, the jejunum was divided into 3-cm sections, from which 1 cm was directly exposed to the Tyrode’s physiological solution and the corresponding hormonal treatments. Each section of the jejunum was carefully washed with Tyrode’s physiological solution. The segment was then everted, as described by Hamilton and Butt (2013). The upper part was secured to a Pasteur pipette with a piece of thread, and the bottom was tied with another. The intestinal segment was filled with 300 µL of Tyrode’s solution containing 5.5 mM of glucose. Glucose measurements required puncturing the everted jejunal sacs to withdraw serosal contents, which compromised sac integrity and precluded further incubation. Therefore, separate and independent sacs were used for the 0- and 30-min measurements. The mucosal side was incubated for 0 min and 30 min with PRL, OT, or PRL + OT diluted in the physiological solution at 37 °C. Glucose concentration was measured on the mucosal side (“out”) using a glucometer CONTOUR®PLUS ELITE (Ascensia Diabetes Care Mexico, S. de R.L. de C.V. Cuautitlan Izcalli, Edomex, Mexico). The device was not formally validated in Tyrode solution using glucose standards; thus, measurements were used for relative comparisons between experimental groups, expressed as % of glucose in the mucosal side.

Glucose concentrations (mg/dL) measured at 0 min from all experimental conditions were pooled and used to establish a common baseline, which was defined as 100% glucose. Glucose values obtained at 30 min for each treatment were normalized to this pooled baseline and expressed as a percentage according to the following equation:

$${Glucose}_{30\;\min}\left(\%\right)=\frac{{Glucose}_{30\;\min}}{{Glucose}_{0\;\min}}\;x\;\;100,$$

where ${Glucose}_{0\;\min}$ represents the mean of the pooled glucose measurements at 0 min.

To estimate glucose uptake (Δ glucose), the percentage value obtained for each treatment at 30 min was subtracted from the baseline (100%), as follows:

$$\Delta \;\text{Glucose }\;\left(\%\right)=100-{Glucose}_{30\;\min}\left(\%\right).$$

This approach allowed comparison of glucose uptake among treatments.

### 2.6 Statistical analysis

The contraction percentage values derived from the treatments with hormones under both cumulative and single administrations were expressed as the mean ± Standard Error of the Mean (SEM) of five biological replicates.

Cumulative concentration-response data were analyzed using a one-way repeated-measures ANOVA, with concentration as the within-subjects factor. When the sphericity assumption was violated, as indicated by Mauchly’s test, Greenhouse–Geisser corrections were applied. Post-hoc pairwise comparisons were performed with the Holm correction. Data are presented as mean ± SEM of five biological replicates.

Individual concentration-response data were analyzed using a one-way ANOVA test for independent samples, and a Tukey post-hoc test to detect differences among groups. Data are presented as mean ± SEM of five biological replicates.

NO analysis data were analyzed using a one-way ANOVA and Tukey post-hoc test to detect differences among groups. Data are presented as mean ± SEM of five biological replicates.

Intestinal everted sacs were analyzed using a one-way ANOVA and Tukey post-hoc test to detect differences among groups. To account for baseline variability, glucose uptake data were normalized and expressed as a percentage of the pooled baseline values obtained at 0 min, which were set to 100%. Comparisons among treatments were then performed using the normalized glucose values measured at 30 min under each experimental condition. Data are presented as mean ± SEM of three biological replicates.

All statistical analyses were performed using JASP software. P-values < 0.05 were considered statistically significant.

## 3 Results

### 3.1 Increasing and cumulative concentrations of PRL- and OT-induced dual effects upon the contraction of the isolated young rat intestine rings

The effects induced by cumulative concentrations (0.01 nM, 0.1 nM, and 1 nM) of PRL and OT were evaluated in KCl-precontracted small intestine rings.

Representative isometric tension recordings of small intestinal segments for each treatment (PRL, OT, and PRL + OT) are provided in the Supplementary Material (Supplementary Figure S1). These traces illustrate the contraction and relaxation profiles obtained under these experimental conditions.

**FIGURE 1:**
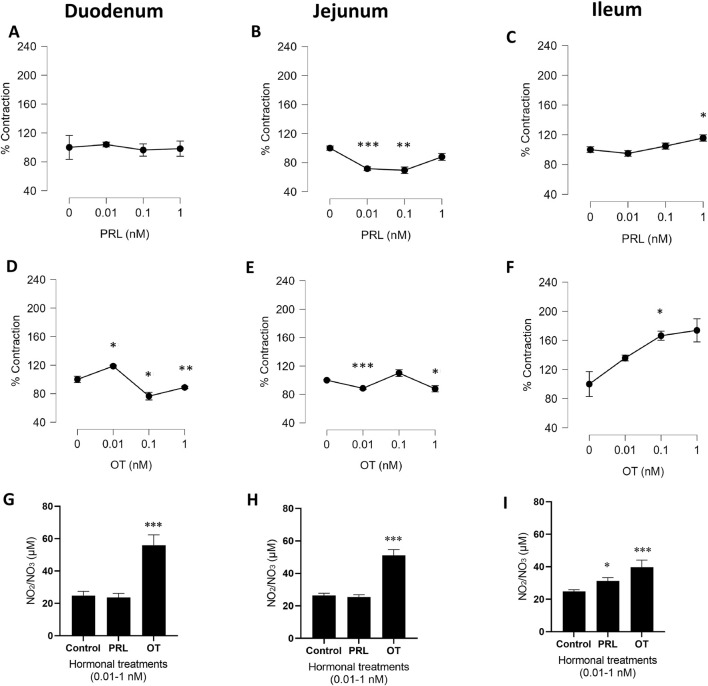
PRL and OT contractile effects in precontracted isolated rings of rat duodenum, jejunum, and ileum. The graphs represent the % of contraction in response to 0.01–1.0 nM of PRL **(A–C)** or OT **(D–F)** compared with the control (0 nM), which is represented by the value of 100% of contraction with KCl. Results are expressed as the mean of the percentages (%) ± SEM. Stabilized responses were measured at 29 min, 49 min, 69 min, and 89 min in experiments using cumulative hormone additions. Following hormone addition, tissues were incubated for 20 min before sample collection. For each time point, nine values were sampled from the stable plateau of the trace, deliberately excluding the transient peaks that occurred immediately after hormone addition. Data were analyzed using repeated-measures ANOVA, with hormone concentration as the within-subject factor and, when applicable, hormone type (PRL vs. OT) as a between-subject factor. NO production expressed as the accumulation of NO_2_/NO_3_ in the physiological solution after treatments **(G–I)**. Values are expressed in µM as mean ± SEM. *P = 0.05, **P = <0.01, ***P < 0.001 vs. the control. The experimental unit was the animal, and n represents the number of animals. n = 5 biological replicates.

From all experiments, the average contraction force was calculated and expressed as a percentage of the 100% KCl-induced contraction. Statistical analyses were performed, and the summarized data are presented in Figures 1A–F, highlighting significant differences between treatments.

The cumulative concentrations of PRL and OT displayed a variety of physiological profiles, like transient contractions (peak contractile responses) or transient relaxations upon the intestinal segments (Supplementary Figure S1). PRL induced a transient contractile effect profile in the duodenum (Supplementary Figure S1A) and jejunum (Supplementary Figure S1B). However, the ileum exhibited a transient relaxing effect (Supplementary Figure S1C). The transient contractile effect induced by PRL in the duodenum was followed by a tension whose magnitude gradually reached 100% of the KCl contraction value (Figure 1A; Supplementary Figure S1A). The jejunum profile induced by PRL exhibited a transient contractile effect (Supplementary Figure S1B). Immediately, the tension was stabilized at values lower than those of the KCl contraction by PRL at concentrations of 0.01 nM (P < 0.001) and 0.1 nM (P < 0.001), as graphed in Figure 1B. In contrast, PRL treatment in the ileum exhibited a transient relaxing effect (Supplementary Figure S1C), followed by the gradual contractile effect graphed in Figure 1C (P < 0.05).

OT in the duodenum induced a contractile response at 0.01 nM (P < 0.05). Increasing the concentration to 0.1 nM reduced contractile activity compared with the previous concentration. At 1 nM, OT again elicited a significant contractile response (P < 0.01), showing a biphasic profile in the stabilized responses (Figure 1D). The representative tension recordings further illustrate the transient contractile and relaxant phases underlying this dual effect (Supplementary Figure S1D). In contrast, in the jejunum, OT caused a transient relaxing effect (Supplementary Figure S1E), followed by a contractile profile graphed in Figure 1E. The ileum segment treated with OT 0.01 nM, 0.1 nM, and 1 nM exhibited a transient relaxing effect (Supplementary Figure S1F), followed by a contractile effect in each concentration tested, as shown in Figure 1F.

Note that Figure 1 does not graphically depict the transient effects caused by PRL and OT in all treated segments; only the stabilized effects are shown.

A contrasting observation was noted when the precontracted jejunum segment was treated with PRL (Supplementary Figure S1B) and OT (Supplementary Figure S1E). The representative jejunal recordings show that the transient contractile effect induced by PRL was opposite to that induced by OT, which caused a transient relaxing effect, suggesting an important role for each hormone in modulating intestinal contractility.

### 3.2 Increasing and cumulative concentrations of PRL intensified the NO production only in the ileum, but OT augmented it in the duodenum, jejunum, and ileum

NO production was measured in the physiological solution (Tyrode’s solution), which contained the intestinal segments treated with the hormones, to determine their potential association with the contractile effect. The increasing and cumulative concentrations of PRL (0.01 nM, 0.1 nM, and 1.0 nM) did not modify the NO production in the duodenum and jejunum segments (Figures 1G,H). However, the hormone induced NO production in the ileum (Figure 1I). Conversely, the increasing and cumulative concentrations of OT (0.01 nM, 0.1 nM, and 1.0 nM) promoted NO production in all segments.

### 3.3 Individual concentrations of PRL- and OT-induced differential effects upon the isolated rat intestine ring contractile function

Considering the transient effects and the consequent differential physiological profiles of PRL and OT on each intestinal segment, we decided to evaluate individual and intermediate concentrations of each hormone and their combination (0.1 nM) in relation to intestinal contractions in each segment compared to the control with KCl (100% of contraction) and their association with NO production.

**FIGURE 2:**
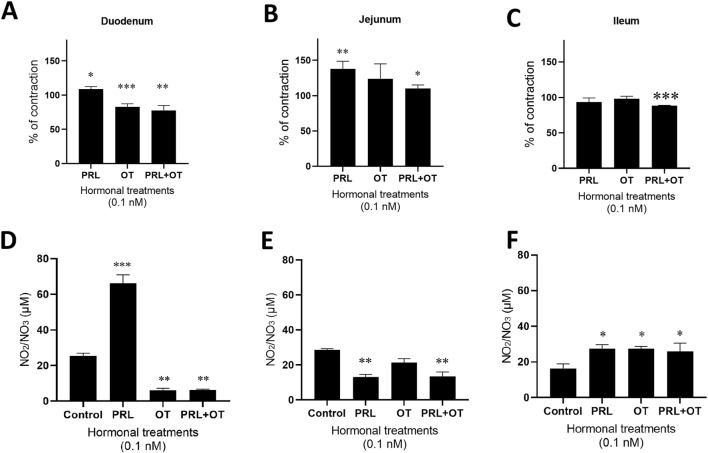
PRL, OT, and their combination (PRL + OT) induced different effects depending on the intestinal segment in precontracted isolated duodenum, jejunum, and ileum rings. The graphs represent the % of contraction in response to a single concentration (0.1 nM) of PRL, OT, or PRL + OT when compared with the control, which is represented by the value of 100% of contraction with KCl **(A–C)**. Results are expressed as the means of the percentages (%) ±SEM. For single-concentration applications, the stabilized response was first evaluated 29 min after KCl-induced precontraction. PRL, OT, or the combination (PRL + OT) was then added, and the tissue was incubated for 20 min. The stabilized effect of each concentration was quantified 20 min after hormone addition (49 min), prior to sample collection. For each time point, nine values were sampled from the stable plateau of the trace, deliberately excluding the transient peaks that occurred immediately after hormone addition. NO production expressed as the accumulation of NO_2_/NO_3_ in the physiological solution after treatments **(D–F)**, and their values are expressed in µM as mean ± SEM. *P = 0.05, **P = <0.01, ***P < 0.001 vs. the control; Statistical differences among groups were analyzed using one-way ANOVA followed by Tukey’s post-hoc test. The experimental unit was the animal, and n represents the number of animals. n = 5 biological replicates.

In the duodenum, we observed that PRL maintained the contraction, but OT decreased it, following the pattern observed in the increasing and cumulative concentrations when 0.1 nM of PRL was administered. However, the combination of PRL and OT decreased the % of contraction, at values similar to those induced by OT alone, showing that the hormonal combination attenuated the contractile effect induced by PRL alone (Figure 2A).

In the jejunum, the administration of PRL induced contraction, and the OT contraction was not different from the control. However, the combination of the hormones again reduced the % of contraction compared to PRL (Figure 2B). In the ileum, the individual and unique administration of PRL or OT was not modified vs. the control with KCl, but the combination of PRL + OT decreased the contraction vs. the control with KCl (Figure 2C).

### 3.4 Individual concentrations of PRL, OT, and the combination of PRL + OT promote differential NO production in the duodenum, jejunum, and ileum

The production of NO was again measured in the physiological solution (Tyrode’s solution) containing the intestinal segments treated with the hormones to evaluate its potential association with the contractile effect.

In the duodenum, PRL increased the production of NO. In contrast, OT reduced this mediator lower than the control value, in the same manner as the treatment with the combination of both hormones (PRL + OT), observing again that the hormonal combination reduced NO production compared to PRL but not to OT (Figure 2D). In the jejunum, in contrast, NO production decreased in the presence of the treatment with PRL, and in the presence of OT, NO production did not change compared to the control. However, combining both hormones (PRL + OT) also decreased the NO levels vs. the control. The effect is comparable to the PRL-induced NO production but not to the OT-induced NO production (Figure 2E). In the ileum, the three treatments, PRL, OT, and the combination (PRL + OT), increased the levels of NO vs. the control (Figure 2F). We did not find a direct association between the contraction profile induced by the hormonal treatments and the NO as a relaxing factor, with exception of the contractile effect induced by PRL associated with a decrement in the NO production in the jejunum (Figures 2B,E), and in the treatment of PRL + OT in the ileum, in which a partial reduction of the contraction was associated with an increase of the NO production (Figures 2C,F), suggesting that NO could not be considered a primary determinant of smooth muscle relaxation in this *ex vivo* model. Rather, NO levels appear to reflect a segment-specific and hormone-dependent modulatory response within a more complex signaling network.

### 3.5 PRL and OT, but not their combination, increase the uptake of glucose in everted intestinal sacs

Because intestinal contractility plays a key role in regulating the exposure of luminal contents to the absorptive epithelium (Chapman et al., 2011), we subsequently examined the effect of hormonal treatments on glucose uptake to explore a possible functional association.

A glucometer was used to determine the glucose concentration (mg/dL) outside the everted intestinal sac, on the mucosal side (out), at time 0 and after 30 min of hormonal treatments. To account for baseline variability, all glucose values obtained at 0 min were pooled and set as 100%, and glucose concentrations measured at 30 min under each experimental condition were subsequently expressed as a percentage of this pooled baseline.

This approach was performed only on the jejunal intestinal sacs exposed to 0.1 nM of PRL, OT, and PRL + OT based on observations showing opposite transient effects of PRL and OT (Supplementary Figures S1B,E). Moreover, this intestinal region is where most glucose absorption occurs (Overduin et al., 2023), and being the largest section of the small intestine (Nzalak et al., 2015), it allows for complete absorption of all treatments within the set period.

**FIGURE 3:**
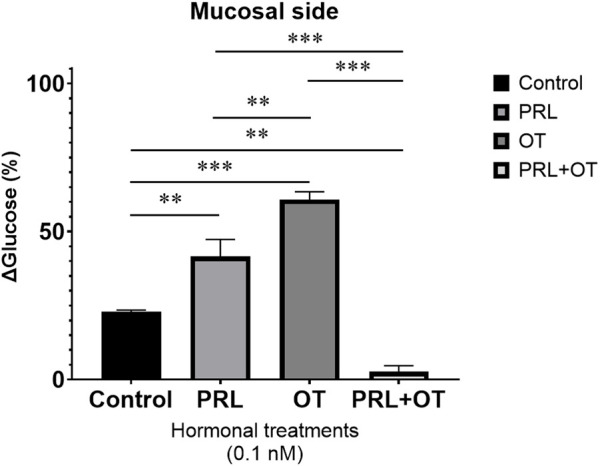
Δ Glucose uptake (%) in everted rat intestinal sacs treated with PRL, OT, or PRL + OT was estimated when everted jejunal sacs were incubated with a single concentration (0.1 nM) of PRL, OT, or their combination (PRL + OT). Glucose concentration in the mucosal solution was measured at 0 min and 30 min using a glucometer. To account for baseline variability, glucose values measured at 0 min from all experimental conditions were pooled and defined as 100%. Glucose values obtained at 30 min were normalized to this pooled baseline and expressed as a percentage. Δ Glucose (%) represents the relative decrease in mucosal glucose concentration after 30 min, calculated as 100 minus the normalized glucose value at 30 min, and was used as a functional estimate of intestinal glucose uptake. Bars represent Δ glucose values measured at 30 min under each treatment condition. Data are presented as mean ± SEM (n = 3 biological replicates) and illustrate relative changes in glucose uptake. *P = 0.05, **P ≤ 0.01, ***P < 0.001 when comparing all groups at 30 min; n = 3 biological replicates.

**FIGURE 4:**
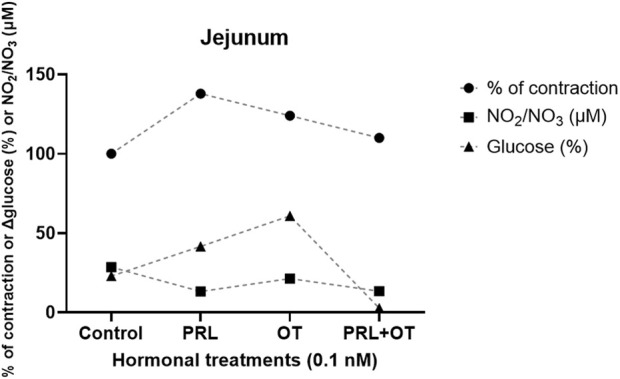
Comparative graphic that represents the main effects induced by PRL, OT, and their combination (PRL + OT) on contractile activity, NO production, and glucose uptake in the jejunum. Isolated jejunal segments were treated with 0.1 nM of each hormone or their combination. Contraction is expressed as a percentage relative to KCl-induced contraction. NO_2_/NO_3_ (μM) levels were determined as an indicator of NO production, and glucose concentrations (%) were measured on the mucosal side at 30 min. PRL and OT individually increased glucose uptake and maintained contractility, while their combination decreased both % of contraction and NO production. Data are expressed as mean values from representative experiments shown in Figures 2B,E, 3.

At 0 min, none of the treatments with OT or PRL + OT altered the basal glucose concentration. However, PRL modified mucosal glucose levels from the initial time point (data not shown). After 30 min, the control condition displayed a decrease in glucose levels, dropping from 100% at 0 min to 77.11% (Δ 22.89%) at 30 min. The PRL treatment reduced glucose from 100% at 0 min to 58.4% (Δ 41.6%) at 30 min (P < 0.01); OT treatment changed glucose levels from 100% at 0 min to 39.16% (Δ 60.84%) at 30 min (P < 0.001). Conversely, the hormone combination (PRL + OT) did not lower the glucose levels (100% at 0 min to 97.37% (Δ 2.63%) at 30 min), modifying the glucose uptake after 30 min of incubation is shown in Figure 3. Figure 4 summarizes the main intestinal effects induced by 0.1 nM PRL, OT, and PRL + OT and their relationship with NO production in the jejunal segments. The results indicate that PRL causes contraction, lowers NO production, and helps uptake glucose from the solution to the mucosal side at 30 min. OT also causes contraction to a lesser degree than PRL, has no effect on NO production, and results in more glucose uptake than PRL. However, the combination of PRL + OT not only maintains contraction but also reduces NO levels and markedly attenuates glucose uptake relative to PRL or OT alone.

Taking this information into account, it is important to mention that in the isolated intestinal ring preparations, both the luminal and serosal surfaces are exposed to the hormone-containing solution, resulting in a global tissue exposure. Consequently, this configuration reflects an integrated tissue-level response rather than selectively modeling luminal or basolateral signaling. Given the reported distribution of the PRLR and OTR in intestinal smooth muscle, enteric neurons, and epithelial cells (Alqudah et al., 2022; García-Caballero et al., 1996; Urtishak et al., 2001; Welch et al., 2009), the contractile and NO-related responses observed in ring preparations likely arise from the combined activation of multiple receptor-expressing cell populations.

Within this experimental framework, the combined treatment with PRL + OT revealed a context-dependent interaction across the evaluated parameters, when interpreted under the distinct effects observed for each hormone administered individually. In the isolated ring preparations, PRL + OT contractile response and was associated with reduced NO levels, suggesting that concurrent activation of PRLR and OTR expressing in the surface of the epithelium or smooth muscle or at enteric neuronal populations may shift the balance toward a contractile profile. Given that both receptors are coupled to intracellular signaling cascades that converge on calcium-dependent and NO-related pathways, their simultaneous stimulation may result in functional crosstalk rather than a simple summation of individual hormonal effects.

In contrast, the everted sac preparation provides a functional model in which hormone exposure is primarily directed to the luminal (apical) surface of the epithelium, where PRLR and OTR expression has been documented in enterocytes and crypt regions (Chen et al., 2015; García-Caballero et al., 1996). Under these conditions, PRL + OT markedly reduced glucose uptake compared with either hormone alone, suggesting that combined hormonal signaling may regulate intestinal glucose handling. Together, these observations support the concept that PRL and OT act as modulatory hormones whose combined actions fine-tune intestinal motor and nutrient handling functions in a context-dependent manner.

## 4 Discussion

In the present study, we investigated the intestinal effects of the hormones PRL, OT, and their combination (PRL + OT) on the contraction of the young rat’s small intestine segments, their relationship to NO production, and their role in the glucose uptake process.

We used an *ex vivo* preparation of juvenile rat small intestine to examine the local, luminal responsiveness of intestinal tissue to PRL and OT, as a physiologically relevant approximation of milk-borne hormonal exposure during early life, rather than modeling the effects of circulating PRL/OT acting systemically on the maternal gastrointestinal tract.

The use of juvenile rat intestine reflects the predominantly postnatal nature of intestinal maturation in altricial species, in which key developmental processes occur during the suckling and weaning periods. Although the human intestine is more mature at birth, both species share conserved sequences of epithelial differentiation, enzyme regulation, and hormonal signaling, supporting the relevance of rodent models for studying early-life intestinal physiology, particularly in the context of prematurity (Drozdowski et al., 2010; Montgomery et al., 1999). In rodents, intestinal exposure to luminal stimuli is a major driver of functional maturation, including the regulation of brush-border enzymes and nutrient transporters (Arévalo Sureda et al., 2016; Castillo et al., 1992). Moreover, PRLR and OTR are expressed in epithelial and enteric neural compartments during early life, supporting the capacity of intestinal tissue to directly integrate milk-borne hormonal signals (Arévalo Sureda et al., 2016; Dena-Beltrán et al., 2025; Liu et al., 2024).

The use of juvenile male rats was intentionally chosen to minimize biological variability associated with the onset of estrous cycle-dependent fluctuations in female sex steroids, which are known to influence intestinal physiology, including motility, barrier function, and related physiological processes (Houdeau, 2016; Mulak, 2014). At juvenile stages, sex-related differences in intestinal physiology are limited, while epithelial maturation and hormonal responsiveness remain active (Arévalo Sureda et al., 2016; Drozdowski et al., 2010). Accordingly, the present model should be interpreted as a tissue-level, mechanistic approach to study intrinsic intestinal hormonal integration, rather than as a direct representation of systemic endocrine regulation during lactation.

The physiological profile induced by these hormones is different in each intestinal segment studied. Moreover, variances exist in the function of the kind of treatment, whether increasing cumulative concentrations or individual concentration treatments.

### 4.1 The PRL role

This multifaceted variety of hormonal actions can be influenced differently by effects on specific small intestine segments, which exhibit a unique anatomy and physiology (Blaker and Irving, 2014).

Consistent with our findings, PRL specifically modulates intestinal contractility in each intestinal segment, as well as NO levels, suggesting a coordinated effect on both contraction and relaxation mechanisms in these segments.

Padmanabha Pillai et al. (1981) demonstrated that PRL-induced contraction in isolated guinea pig ileum segments, similar to that caused by acetylcholine (ACh), a contractile agent in the intestine. This process is mediated by a cholinergic mechanism involving muscarinic receptors because adding atropine, a muscarinic receptor blocker, in the presence of PRL, reduced the contractile effects. Activation of muscarinic receptor isoforms (such as M1, M3, or M5) by ACh belongs to the family of G protein-coupled receptors, which activate phospholipase C, promoting the formation of diacylglycerol and inositol 1,4,5-trisphosphate. These, in turn, activate protein kinase C (PKC), mobilizing intracellular calcium either directly or via store-operated calcium entry (SOCE), thereby stimulating smooth muscle contraction (Uwada et al., 2023).

In this context, it is possible that the fine regulation and interaction of PRL with its intestinal receptor (Radojkovic et al., 2024; Urtishak et al., 2001) could crosstalk with the complex activation of muscarinic receptors by activating contractile responses. This may potentiate, sustain, or reduce the contractile effect in the duodenum, jejunum, and ileum treated with increasing and cumulative concentrations, or with a single PRL concentration (0.1 nM).

PRL is a crucial regulator of systemic calcium balance. In the small intestine, enhanced calcium mobilization was observed to promote permeation and absorption in six-month-old pigs (Dolinska et al., 2011), suggesting a predominant contractile effect in the studied isolated intestinal segments, consistent with other studies, where PRL can induce a contractile effect through a cholinomimetic mechanism in the isolated guinea pig ileum rings (Gopalakrishnan et al., 1981).

It is also reported that PRL binds to its PRL receptors in epithelia, such as the endothelium (Gonzalez et al., 2015; Radojkovic et al., 2024; Rosas-Hernandez et al., 2013). When increasing and cumulative concentrations of PRL were administered, they exerted dual effects on vascular tone in isolated rat aortic rings, inducing a contraction followed by relaxation, which was NO-independent and endothelium-dependent. In contrast, the single administration of PRL promoted a PGI_2-_ dependent relaxation. In the duodenum epithelium, PRL binds to its receptor and stimulates calcium absorption (Radojkovic et al., 2024).

To these observations, the maintenance and stimulation of the contractile effect induced by increasing and cumulative concentrations of PRL in the duodenum showed no modification of NO compared to the control. However, in jejunum under this kind of administration, the relaxation at the concentrations of 0.01 nM and 0.1 nM and recovery of the contraction at the control level were not associated with the production of NO, suggesting the participation of another kind of mediators, for instance, arachidonic acid derivatives or ACh, the main contractile neurotransmitter in the GI tract (Gonzalez et al., 2015; Padmanabha Pillai et al., 1981; Coletto et al., 2019). In contrast, in the ileum, even prior to the establishment of the contractile effect induced by each increasing concentration of PRL, a marked transient relaxant response was observed (Supplementary Figure S1), followed by contraction. This early relaxation may be associated with a predominant production of NO, which could transiently override or obscure the contribution of other mediators not quantified in this study.

In this sense, a bolus of increasing concentrations of PRL stimulated eNOS activity in isolated hearts of guinea pigs, and then the NO production induced vasorelaxation. This action was blocked by vasoinhibins (Vi), a proteolytic fragment derived from PRL, inducing opposite effects on heart function, such as vasoconstriction due to the inhibition of NO production, as Vi blocked eNOS activity (González et al., 2008).

It is possible that this dual profile induced by PRL in the intestinal segments could partly explain these combined effects as a function of the evaluation time, in which the presence of PRL and a hypothetical generation of PRL fragments (or Vi) exert opposite effects: contraction and relaxation, collaborating in the maintenance and homeostasis of the intestinal motility.

Moreover, it has been shown in anesthetized pigs that the coronary, mesenteric, renal, and iliac vasoconstriction caused by PRL infusion through the blockage of the vasodilatory β_2_-adrenergic receptor-mediated effect is related to the NO intracellular pathway (Molinari et al., 2007).

These vascular findings are relevant because they suggest that PRL could exert, in part, similar regulatory mechanisms in other muscle tissues, like the intestine.

The present study identified a common set of extensive effects induced by PRL in regulating intestinal function. For instance, we found that successive concentrations of PRL induced a transient effect, resulting in duodenum contraction, jejunum relaxation, and ileum contraction. However, PRL did not induce increases in NO production compared with the control, except for the ileum. These data suggest that PRL may function directly or indirectly through a mediator that modifies the degree of contraction following successive hormone administrations. According to these findings, chronic administration of PRL was reported to increase the response to contractile agents (KCl and ACh) in the smooth muscle cells of the seminal vesicles (Firdolas et al., 2013). Moreover, the inhibition of PRL with bromocriptine decreased this contractile response (Firdolas et al., 2013), suggesting and reinforcing the fact that PRL may regulate smooth muscle contraction of biological conduits.

Recently, Dena-Beltrán et al. (2025) demonstrated that PRL modulates neonatal intestinal epithelial cells. In intestinal spheroids derived from neonatal mouse epithelium, PRL promoted cell proliferation and activated the AKT and ERK1/2 signaling pathways. *In vivo*, the absence of PRL receptor signaling accelerated intestinal maturation, as evidenced by morphological changes and reduced expression of neonatal epithelial markers, including lactase activity. These findings point out that PRL contributes to maintaining the neonatal intestinal epithelial phenotype. Collectively, this work supports the concept that milk-derived PRL may function as a maternal regulatory signal during early intestinal development within the framework of neonatal intestinal physiology, including key aspects as nutrient handling and epithelial maturation. In addition, one patent describes the use of PRL as a food ingredient with potential therapeutic effects, supporting the emerging view of PRL not only as a physiological hormone but also as a candidate bioactive compound in the regulation of intestinal function (Naim, 2010).

### 4.2 The OT role

Regarding the actions induced by OT on the intestinal segment’s contraction, most of the observations during the recordings (Supplementary Figure S1) indicated that, in response to cumulative concentrations of OT, each intestinal segment exhibited an initial transient relaxation followed by a sustained contraction, comparable to the KCl control in the duodenum and jejunum, and exceeding it in the ileum (Figure 1).

NO production, during cumulative administrations of OT, increased approximately two times in the jejunum and ileum and approximately three times in the duodenum compared to the control.

However, the single administration of OT showed a differential production of NO, with lower levels in the duodenum, no changes in the jejunum, and higher levels in the ileum vs. the control. However, NO production was not always directly correlated with the observed contractile profile.

In this context, in the duodenum (Figure 2D), we observed a reduction in basal NO_2_^−^/NO_3_^−^ levels following OT administration, suggesting a potential interaction between OT and NO-related species beyond modulation of NO production. OT contains a cysteine residue that may undergo S-nitrosation, a chemical modification mediated by NO or reactive nitrogen species. Roy et al. (2007) demonstrated that OT can be S-nitrosated under defined reaction conditions, resulting in structural and functional alterations. Although the *ex vivo* intestinal preparation used in the present study does not allow direct assessment of S-nitrosation reactions, limited chemical interactions between OT and reactive nitrogen species cannot be excluded and may contribute, at least in part, to the attenuation of pre-established NO_2_^−^/NO_3_^−^ levels observed here. In support of this notion, OT has been reported to reduce basal NO levels in sepsis-sensitized macrophages (Oliveira-Pelegrin et al., 2013), suggesting that OT may modulate NO-related signaling under basal conditions across different biological systems.

In addition, the effects induced by OT in various biological experimental models exert multiple actions due to the extensive signaling pathways activated by this hormone. These actions also depend on factors such as concentration, exposure time, route of administration, experimental conditions, and models (Abood and Ahmed, 2012; Che et al., 2012; Lv et al., 2010). OT induces eNOS phosphorylation and vasorelaxation through activation of the OTR/PI3K/Akt pathway in human umbilical vein endothelial cells (HUVECs) *in vitro* (Xu et al., 2022). In the rat aorta, OT displays a biphasic response. *Ex vivo* incubation of OT (10 nM) for 5 min evokes vasoconstriction in aortic rings precontracted with 30 nM norepinephrine, while cumulative concentrations of OT (10^11^–10^7^ M) reveal that low doses (10^9^–10^8^ M) induce vasorelaxation through the PI3K/Akt/eNOS–NO pathway, whereas a higher concentration (10^7^ M) elicits vasoconstriction via OTR/V1aR–ERK1/2 signaling (Xu et al., 2022).

At concentrations that induce penile erection and yawning, OT increases NO production in the paraventricular nucleus of the hypothalamus in male rats by activating its own receptors in that region (Li et al., 2015; Melis et al., 1997). Another study shows that in the rat proximal colon, OT can inhibit spontaneous contractions and enhance electrically evoked noradrenergic, noncholinergic (NANC) relaxation in longitudinal smooth muscle *in vitro*, effects that were associated with NO signaling within the enteric nervous system (Wang et al., 2016). Similar relaxing effects were observed in the present study during cumulative OT concentration–response experiments, particularly at 0.1 and 1 nM in the duodenum and at 0.01 and 1 nM in the jejunum, consistent with increased NO production. In conscious starving dogs, OT administration modulates muscle tone in the stomach and intestines, inhibiting both peristaltic contractions of the stomach and small intestine. A similar effect was observed in muscle strips isolated from the guinea pig stomach, where spontaneous contractions were blocked (Milenov, 1979). Likewise, OT at 0.1 U/L and 1 U/L, but not at 0.01 U/L, inhibited spontaneous contractile activity in the proximal colonic circular and longitudinal smooth muscle in NO-independent rabbits (Xie, 2003).

Recently, Alqudah et al. (2022) demonstrated in an organ bath preparation that exogenous OT (10^−9^–10^−5^M) directly stimulates contraction of rat gastric circular smooth muscle via activation of the OTR, which in turn engages the PLC pathway, leading to IP_3_ generation, intracellular calcium mobilization, and subsequent smooth muscle contraction. Pharmacological inhibition confirmed pathway involvement: pre-incubation with atosiban (an OTR antagonist) abolished OT-induced contraction. Likewise, U73122 (a PLC inhibitor), Xestospongin C (an IP_3_ receptor antagonist), and verapamil (a voltage-dependent calcium channel blocker) significantly attenuated the contractile response. Chelation of intracellular calcium with BAPTA-AM and pharmacological inhibition of calcium/calmodulin-dependent protein kinase kinase (CaMKK) by STO-609 also suppressed OT-induced contraction, further supporting the contribution of calcium-dependent signaling to OTR-mediated regulation of gastrointestinal motility.

In agreement with this evidence, our work finds that, particularly in the ileum, increasing and cumulative concentrations of OT promote a contractile effect, and the single concentration of OT (0.1 nM) maintains the contractile effect in the jejunum and ileum. In addition, at the concentration of 10^-9^ M OT used by Alqudah et al. (2022) and in our work, this hormone also stimulated an important contraction observed in the ileum segment (Figure 1F), suggesting that the effects induced by OT in the gastrointestinal tract can share several actions upon their smooth muscle cells destined to regulate the muscle tone, motility, conferring a fine balance on the contraction or relaxation modulated by OT. Other studies have shown that OT directly regulates colon function, influencing intestinal permeability, gastrointestinal transit, and protecting against colitis-induced damage (Welch et al., 2014). It has been shown to have anti-inflammatory effects (İşeri et al., 2005) and can reduce oxidative injury of the colon (Welch et al., 2014).

The OT gastrointestinal effects also influence other tissues and functions, suggesting broader biological roles. OT stimulates the release of PGI_2_ in the pregnant rat myometrium, which has anti-aggregatory and vasodilatory effects (Williams and El Tahir, 1980). In rabbits, OT affects the permeability of the mammary epithelium, leading to changes in the milk’s sodium, chloride, potassium, lactose, fat, and protein levels, which may support nutrient absorption in the small intestine (Linzell et al., 1975). These multifaceted actions support the growing interest in OT as a bioactive molecule with relevance beyond its classical roles, acting as a regulator of intestinal function, including motility, smooth muscle contraction, and nutrient absorption.

In the present work, OT exerted a dual effect, in which the transient relaxation could induce NO production, suggesting that an isoform of OTR-induced NO, and subsequently, the contractile effect could be caused by another OTR isoform. Although the isoforms present in the small intestine have not been fully characterized, OTR isoform diversity has been reported in other tissues, such as the uterus of prairie voles (Danoff et al., 2023). However, the potential involvement of distinct OTR isoforms in the dual actions of OT on intestinal motility remains to be elucidated, including their identification and their possible functional relationship during lactation.

### 4.3 The PRL + OT role

An important finding was the opposite effects of PRL and OT, which were more evident in the jejunum (Supplementary Figure S1). When each increasing and cumulative administration of PRL induced a transient contractile effect, the same kind of administration of OT induced a transient relaxing effect.

In this context, and based on previous evidence, it is important to consider that little is known about their intestinal actions. Moreover, few studies address the combined effects of these hormones on small intestine contractility, their relationship with nutrient handling (such as glucose), and their possible involvement in NO production. We decided to evaluate the combination using an equimolar mixture of PRL + OT.

This mixture noticeably and predominantly decreased the contractile effect compared to PRL alone in the duodenum, jejunum, and ileum. In the ileum, this decrease also contrasted with the effect of OT.

Further, the non-additive effect observed when PRL and OT were administered together suggests that OT may exert a modulatory rather than a purely stimulatory role on PRL-mediated intestinal glucose uptake. This concept is consistent with evidence from other endocrine systems in which OT functions as a homeostatic regulator. For example, central OT administration attenuates stress-induced activation of the hypothalamus–pituitary–adrenal axis, reducing adrenocorticotropic hormone and corticosterone release without abolishing basal hormonal activity (Windle et al., 2004). Such findings support the notion that OT can constrain or fine-tune hormonal responses under conditions of combined hormonal signaling. In this context, the attenuation of glucose uptake observed with PRL + OT treatment may reflect an intrinsic integrative mechanism of the intestinal tissue, preventing excessive or dysregulated glucose mobilization.

Regarding NO production, this mixture decreased NO production compared to the PRL alone in the duodenum. In the jejunum, that mixture decreased NO production compared to OT at levels lower than the respective control. However, in the ileum, the hormonal mixture maintained NO levels to those observed with PRL and OT alone, which were higher than the control values.

Although changes in NO production were detected following PRL, OT, and PRL + OT treatments, these variations did not consistently correlate with the direction or magnitude of the contractile responses across intestinal segments. Therefore, based on the present data, NO cannot be considered a primary determinant of smooth muscle relaxation in this *ex vivo* model. Rather, NO levels appear to reflect a segment-specific and hormone-dependent regulatory response within a more complex signaling network. Importantly, the present study does not allow discrimination between NO-dependent and NO-independent mechanisms, as pharmacological inhibition of NO synthesis was not performed. Future studies using NOS inhibitors will be necessary to directly address the contribution of NO signaling to PRL- and OT-induced intestinal effects.

Taken together, these findings suggest a functional interaction between PRL and OT in the modulation of intestinal contractility, where OT may attenuate PRL-induced effects, pointing to potential crosstalk between their respective receptors and downstream signaling pathways. Given that both PRL (Dena-Beltrán et al., 2025) and OT (Welch et al., 2014) receptors are coupled to intracellular cascades, they could share common intermediates, and it is plausible that concurrent stimulation could result in synergistic, antagonistic, or modulatory actions. Such interactions may involve receptor cross-activation, inhibition, or desensitization, ultimately modifying the physiological responses observed when each hormone is administered individually.

### 4.4 Hormonal regulation of the intestinal glucose uptake

Based on the well-established cooperative roles of OT and PRL during breastfeeding, where PRL sustains the continuous milk production (Al-Chalabi et al., 2023), and OT promotes milk ejection (Pillay and Davis, 2023), ensuring an efficient and continuous supply to the infant, it is plausible that a similarly coordinated regulatory system may operate in the small intestine to optimize nutrient absorption. It is possible that a fine regulatory system could be maintained in the small intestine to favor the efficient absorption of all the nutrients. An example of that action could be revealed with the use of glucose uptake through the technique of the everted rat jejunal intestine sacs, after 30 min of incubation, OT produced the highest Δ glucose (%), reflecting a greater reduction in mucosal glucose concentration compared with PRL. In contrast, the combined PRL + OT treatment markedly reduced Δ glucose, suggesting a non-additive regulatory interaction.

These data are consistent with the effects induced by OT on glucose uptake in muscle tissues. In skeletal muscle cells, OT activates intracellular Ca^2+^ signaling, which in turn stimulates the CaMKK–AMPK pathway, leading to increased glucose uptake (Lee et al., 2008). Similarly, in neonatal cardiomyocytes, OT facilitates glucose uptake through the PI3K/Akt pathway, promoting GLUT4 translocation to the plasma membrane (Florian et al., 2010). Evidence also suggests that OT improves glucose and lipid metabolism in skeletal muscle and adipose tissue, contributing to insulin sensitivity (Ding et al., 2019). In this context, the reduction of extracellular glucose concentrations observed in our intestinal preparations could be partly explained by an OT-induced stimulation of glucose uptake in the jejunum mucosal side.

In the case of PRL, *in vivo* studies reported that PRL administration increases intestinal absorption of glucose and nutrients in rats, although these effects are less evident under direct *in vitro* conditions (Mainoya, 1975). More recent work indicates that PRL contributes to maintaining the neonatal enterocyte phenotype during lactation, supporting a role in intestinal epithelial function (Dena-Beltrán et al., 2025). However, current evidence for a direct regulation of enterocyte glucose transporters by PRL is scarce, and its effects on glucose uptake in the intestine remain largely unexplored.

In the present study, glucose uptake was assessed using a glucometer as a practical approach to monitor relative changes in glucose concentration in the everted intestinal sac preparation. While this method allows the detection of overall alterations in glucose uptake, the specific molecular mechanisms underlying the hormonal actions were not addressed in the present work. Notably, the combined equimolar concentration of PRL and OT (0.1 nM) reduced intestinal glucose handling compared with each hormone administered alone, resulting in glucose levels that remained close to baseline values. Such regulation could contribute to the fine-tuning of nutrient handling during developmental stages, such as lactation and breastfeeding, although this hypothesis requires further experimental validation.

When PRL and OT were administered simultaneously, intestinal contraction decreased. We hypothesize that reduced motility, reflecting decreased contractile activity at experimental hormone concentrations, may compromise intestinal transit and limit glucose uptake, ultimately affecting nutrient availability and energy supply. Even when PRL concentrations were lower than those reported in other experimental studies, they induced measurable effects on intestinal contractility, NO production, and glucose uptake, supporting the physiological relevance of low hormone concentrations in intestinal development and maintenance of intestinal function.

Moreover, the present *ex vivo* approach does not allow direct extrapolation to the *in vivo* situation. It provides a controlled and physiologically relevant framework to identify tissue-hormone actions and to generate testable hypotheses for future *in vivo* and mechanistic studies. In this context, our findings gain relevance when considering early-life nutritional exposures, as physiological levels of PRL and OT in breast milk are tightly regulated, preventing adverse effects on intestinal function. The observed young intestinal sensitivity to these hormones underscores the importance of carefully considering hormone concentrations if PRL or OT were ever to be incorporated into infant formulas or explored as therapeutic agents for digestive disorders, to avoid unintended alterations in nutrient absorption.

### 4.5 Additional considerations

The present study was conducted using an *ex vivo* intestinal model, which allows controlled evaluation of direct tissue-level responses to PRL, OT, and their combination but does not fully replicate the complexity of the *in vivo* intestinal environment. Juvenile rat small intestine was selected not because it is structurally identical to neonatal intestine, but because it preserves the overall organization of the intestinal epithelium and key functional maturation processes relevant to early-life physiology within a more stable and experimentally robust tissue (Drozdowski et al., 2010).

In line with general descriptions of intestinal development and mucosal adaptation, many functionally relevant processes are established early in life and progressively refined during postnatal maturation (Drozdowski et al., 2010). Importantly, the juvenile intestine provides improved tissue viability, mechanical integrity, and reproducibility under *ex vivo* conditions, which are technically challenging to achieve using neonatal tissue. In this context, the experimental design presupposes the presence of defined tissue concentrations of these hormones, while the extent to which intact PRL and OT reach the intestinal lumen during breastfeeding remains incompletely characterized, rendering digestive stability and bioavailability important limitations of the present approach. Despite these constraints, measurable functional responses were observed even at low hormone concentrations, particularly for PRL, indicating high intestinal sensitivity under these experimental conditions and supporting the physiological relevance of the model.

## Data Availability

The original contributions presented in the study are included in the article/Supplementary Material; further inquiries can be directed to the corresponding author.
