# Supplementary material for: Prolactin and oxytocin as modulators of intestinal contractility and glucose uptake
Source: Front Physiol. 2026 Feb 26;17:1703887. doi: 10.3389/fphys.2026.1703887 (PMC12979113; doi:10.3389/fphys.2026.1703887)
Supplement: Supplementary file 1 [file DataSheet1.pdf]

Prolactin and oxytocin as modulators of intestinal contractility and glucose uptake

Perla Alejandra Figueroa-Carrasco, Aída Jimena Velarde-Salcedo, Carmen Gonzalez*

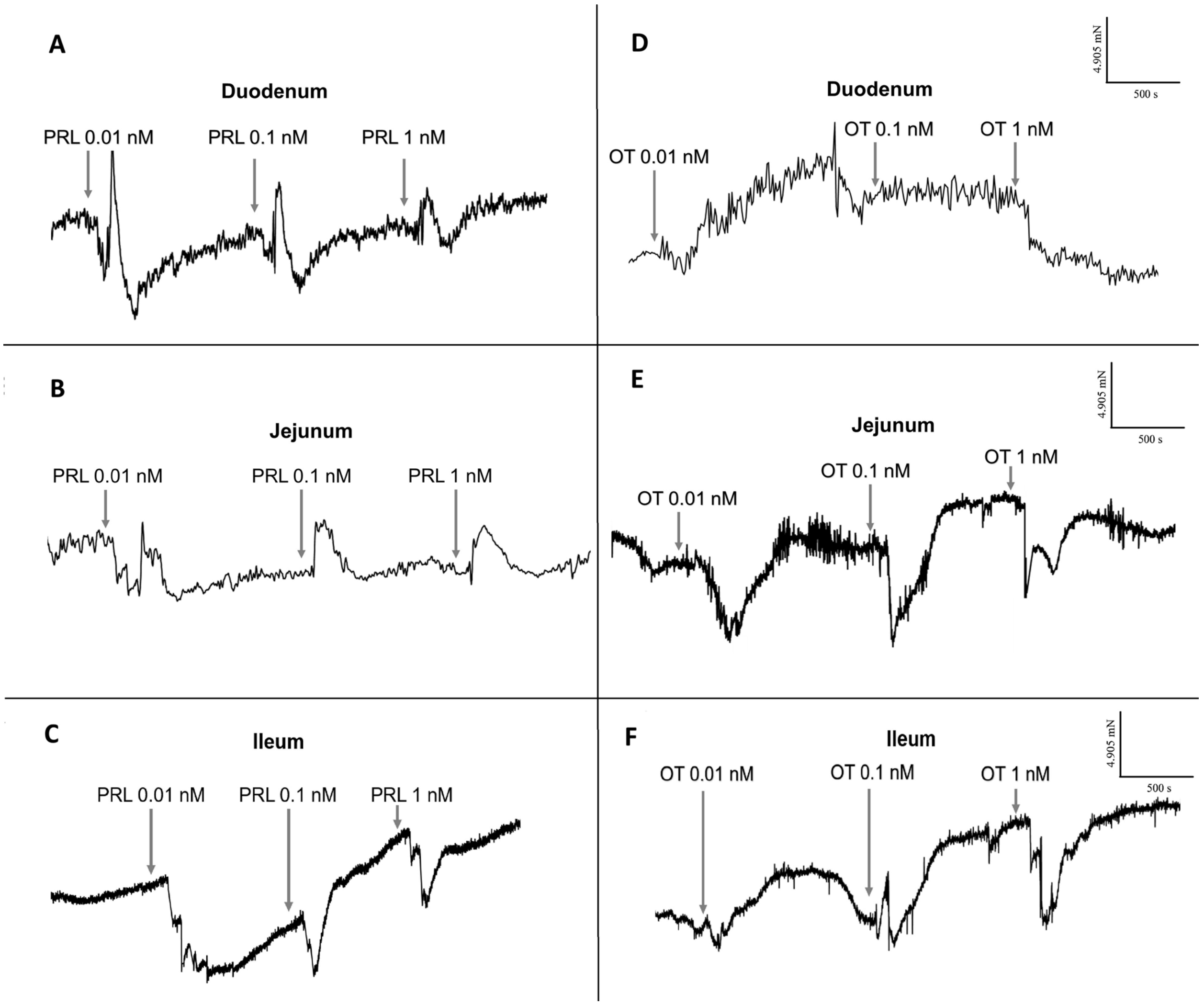

Figure S1. PRL and OT induced a dual effect in precontracted isolated rat duodenum, jejunum, and ileum rings. Representative physiological recordings of tension (mN) in response to cumulative concentrations (0.01-1.0 nM) of PRL (A, B, C) and OT (D, E, F). n=5 animals per group.
